# Mathematical Analysis of Hall Effect on Transient Hartman Flow about a Rotating Horizontal Permeable Surface in a Porous Medium under Inclined Magnetic Field

**DOI:** 10.1155/2014/765140

**Published:** 2014-11-23

**Authors:** M. Suresh, A. Manglik

**Affiliations:** Theoretical and Computational Geophysics Division, CSIR-National Geophysical Research Institute, Uppal Road, Hyderabad 500007, India

## Abstract

This paper proposes the exact solution for unsteady flow of a viscous incompressible electrically conducting fluid past a impulsively started infinite horizontal surface which is rotating with an angular velocity embedded in a saturated porous medium under the influence of strong magnetic field with hall effect. Our study focuses on the change of direction of the external magnetic field on the flow system which leads to change in the flow behavior and skin frictional forces at the boundary. Systems of flow equations are solved using Laplace transform technique. The impacts of control parameters Hartman number, rotation of the system, hall effect, inclination of the magnetic field, and Darcy number on primary and secondary velocities are shown graphically, skin friction at horizontal boundary in tabular form. For validating our results, in the absence of permeability of the porous medium and inclination of the magnetic field the results are in good agreement with the published results.

## 1. Introduction

The problem of convection resulting from the flow over a horizontal plate provides probably one of the most fundamental problems of MHD convection in Earth, astrophysical and solar physics with Layered model, and is thus of considerable theoretical and practical interest. In spite of the fact that a great number of analytical and numerical results are available for these problems it still continues to be a topic of vital importance. MHD Couette flow in presence of hall currents and inclined magnetic field appears in these reviewed papers; Katagiri [1] investigated Couette flow in the presence of transverse magnetic field, Jana and Datta [2] analyzed by imposing Hall current and Ghosh [3] examined the problem with inclined magnetic field. Krishna et al. [4] discussed MHD Couette flow through a porous medium; Rana et al. [5] considered Burger's fluid between two parallel electrically insulating planes with hall effect and this flow problem is further examined through porous medium past vertical and rotating disks in [1–9]. Steady and unsteady MHD flow past a horizontal plate appears in [10–16] presented analytical solutions using the methods similarity variables, Laplace transform technique, and homotopy analysis.

**Figure 1:**
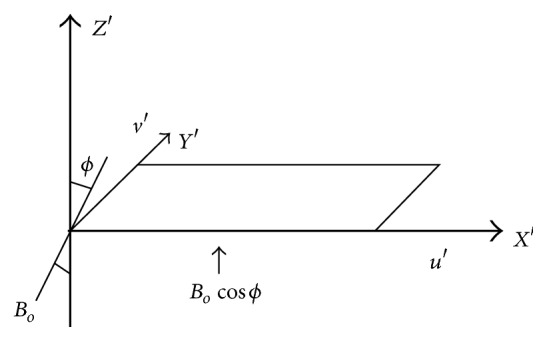
Physical model and coordinate system.

Rich [17] seems to be the first to consider the case study of fluid flow through a porous medium in inclined plates both analytically and experimentally by replacing the gravity acceleration by its tangential component *g*cos⁡*ϕ*, in the buoyancy force term of the momentum equation where *ϕ* is the angle of inclination of the plate from the vertical direction. Later Jiin-Yuh and Wen-Jeng [18] discussed the vortex instability results for small angles of inclination from the horizontal plate. Seth et al. [19, 20] and Beg et al. [21] examined unsteady rotating flow with hall current imposing an inclined magnetic field. Rao et al. [22] gave an FEM solution for the problem of hall effect on MHD unsteady rotating fluid flow past a horizontal plate. We propose an analytical solution for the problem of unsteady flow past a horizontal rotating surface through a porous medium in the presence of strong inclined magnetic field with hall effect and the physical model shown in Figure 1.

## 2. Formulation of the Basic Equations

In this mathematical model, we consider the transient flow of viscous electrically conducting fluid whose thermophysical and chemical properties are assumed to be constant with negligible buoyancy force in the system, through a porous medium past a suddenly started electrically nonconducting infinite horizontal rotating permeable plate boundary in the presence of uniform magnetic field in a direction which makes an angle *ϕ* with the positive direction of normal to the boundary. In this model, no polarized voltages should exist and the fluid permeated by magnetic field is strong enough to count the hall currents and is neglecting the induced magnetic field assuming magnetic Reynolds number is small for liquid metal and ionized fluids. Initially, as the fluid and plate boundary are rotating with a constant angular velocity *Ω*′ and consider in such a way which makes to neglect the centripetal acceleration in Navior-Stoke's equation; also in small interval of time the plate boundary is moved suddenly with certain velocity after which it maintains a constant velocity relative to the system. Choose the Cartesian coordinate system (*X*′, *Y*′, *Z*′), the infinite plane *z*′ = 0 as *X*-axis in horizontal direction of the plate velocity and *Y*-axis is perpendicular to horizontal direction of the plate; clearly *z*′ = 0 represents the infinite plate with *Z*-axis being normal to the plate. Let *q* = (*u*′, *v*′, *w*′) and *J* = (*J*_*x*_′, *J*_*y*_′, *J*_*z*_′) be the velocity of the fluid and the current density vector with magnetic field *B*. Generalized basic equations of the flow derived from the conservation laws, Gauss law of magnetism, Kirchhoff's first law, Continuity, Navior Stokes, and Generalized Ohm's law to represent the flow of viscous incompressible electrically conducting fluid in presence of strong magnetic field embedded in a porous medium. Consider

(1) ∇·B=0,∇·J=0,∇·q=0,∂q∂t′+(q·∇)q+2Ω′×q+Ω′×Ω′×r′  =−∇p+μeσJ×B+υ∇2q−υk1q,J+ωeτeBoJ×Bq=σE+q×B+∇pee∗ne.

Mathematical model assumptions of the problem are

(2) ∂w′∂z′=0⟶w′=−wo′; ∂p′∂z′=0; m=ωeτe;∂Jz′∂z′=0⟶Jz′=0⟹Jx′=σBocos⁡ϕv′+mcos⁡ϕu′1+m2;Jx′=σBocos⁡ϕmcos⁡ϕv′−u′1+m2.

The governing equations of the problem with relevant boundary conditions derived from the assumptions equation (2), and (1) can be written as

(3) ∂u′∂t′−w0′∂u′∂z′−2Ω′v′  =υ∂2u′∂z′2+σBo2cos⁡2ϕmcos⁡ϕv′−u′ρ1+m2cos⁡2ϕ−υk1u′,∂v′∂t′−w0′∂v′∂z′+2Ω′u′  =υ∂2v′∂z′2−σBo2cos⁡2ϕmcos⁡ϕu′+v′ρ1+m2cos⁡2ϕ−υk1v′,

(4) $$\begin{array}{c} u^{\prime }\left(z^{\prime },0\right)=v^{\prime }\left(z^{\prime },0\right)=0;\;\;u^{\prime }\left(0,t^{\prime }\right)=U_{o}^{\prime },\\ v^{\prime }\left(0,t^{\prime }\right)=0;\;\;u^{\prime }\left(\infty ,t^{\prime }\right)=v^{\prime }\left(\infty ,t^{\prime }\right)=0, \end{array}$$

where *t*′, *w*_*o*_′, *Ω*′, *k*_1_, *B*_*o*_, *ϕ*, *E*, *σ*, *p*, *ρ*, *υ*, *e*, *ω*_*e*_, *τ*_*e*_, *p*_*e*_, *m*, and *n*_*e*_ are small interval of time, suction velocity, rotation velocity of the system, porous medium permeability, strength applied magnetic field, inclination of the magnetic field with the normal to the plate, electric field intensity, electrical conductivity, fluid pressure, fluid density, kinematic viscosity, electric charge, cyclotron frequency, collision time, pressure of the electron, and number representing electron density.

Introducing the following dimensionless variables

(5) $$\begin{array}{c} z=\frac{w_{o}^{\prime }z^{\prime }}{\upsilon },\;\;t=\frac{w_{o}^{\prime 2}t^{\prime }}{\upsilon },\;\;u=\frac{u^{\prime }}{U_{o}^{\prime }},\;\;v=\frac{v^{\prime }}{U_{o}^{\prime }} \end{array}$$

in (3) and (4) we get

(6) $$\begin{array}{c} \frac{\partial u}{\partial t}-\frac{\partial u}{\partial z}-\Omega v=\frac{\partial^{2}u}{\partial z^{2}}+\frac{M\cos^{2}\phi \left(m\cos\phi v-u\right)}{\left(1+m^{2}\cos^{2}\phi \right)}-Du,\\ \frac{\partial v}{\partial t}-\frac{\partial v}{\partial z}+\Omega u=\frac{\partial^{2}v}{\partial z^{2}}+\frac{M\cos^{2}\phi \left(m\cos\phi v-u\right)}{\left(1+m^{2}\cos^{2}\phi \right)}-Dv, \end{array}$$

(7) $$\begin{array}{c} u\left(z,0\right)=v\left(z,0\right)=0;\;\;u\left(0,t\right)=1,\;\;v\left(0,t\right)=0;\\ u\left(\infty ,t\right)=v\left(\infty ,t\right)=0. \end{array}$$

Combining the equations in (6) using the complex velocity *F* = *u* + *iv*, we get momentum equation in complex velocity

(8) ∂F∂t−∂2F∂z2−∂F∂z+gF=0,Fz,0=0; F0,t=1, F∞,t=0,

where *D* = *υ* ^2^/*k*_1_ *U*_*o*_ *w*_*o*_^′2^ (Darcy parameter), *M* = *σB*_*o*_^2^ *υ*/*ρw*_*o*_^′2^ (Hartman number), *η* = *f*(*z*, *t*), *Ω* = *Ω*′*υ*/*U*_*o*_ *w*_*o*_^′2^ (rotation parameter), and *g* = (*M*cos⁡^2^ *ϕ*/(1 + *m* ^2^cos⁡^2^ *ϕ*) + *D*) + *i*(*Mm*cos⁡^3^ *ϕ*/(1 + *m* ^2^cos⁡^2^ *ϕ*) + *Ω*).

## 3. Analytical Solution

Using finite element method, Rao et al. [22] solved the problem without considering inclined direction of the magnetic field and porous medium of the flow. But we solve the problem by considering the above effects analytically using Laplace transform, into ordinary differential equations

(9) d2F−dz2+dF−dz−g+sF−=0,F−0,s=1s;  F−∞,s=0,

(10) $$\begin{array}{c} \overset{-}{F}=e^{-0.5z}\left[K_{1}e^{\left(\sqrt{1+4g+4s}\right)z}+K_{2}e^{-\left(\sqrt{1+4g+4s}\right)z}\right], \end{array}$$

where *K*_1_ = 0, *K*_2_ = 1/*s* are constants using boundary conditions.

Using the Laplace inverse properties

(11) $$\begin{array}{c} f\left(t\right)=\mathrm{erfc}\left(\frac{a}{2\sqrt{t}}\right),\;t>0,\;L\left[f\left(t\right)\right]=\frac{1}{s}e^{-a\sqrt{s}}. \end{array}$$

Applying inverse-Laplace to (10) and on simplifying we get

(12) F=0.5e−ηte2ηgterfc⁡η+gt+e−2ηgterfc⁡η−gt.

The expression for viscous shear stresses at the plate boundary is

(13) τ=τx+iτy=∂F∂ηη=0=∂u∂ηη=0+i∂v∂ηη=0=1+1πe−gt+gerf⁡gt,

where (*τ*_*x*_, *τ*_*y*_) are primary and secondary shear stresses, *s* is Laplace constant, and *η* = *f*(*z*, *t*).

## 4. Results and Discussions

**Figure 2:**
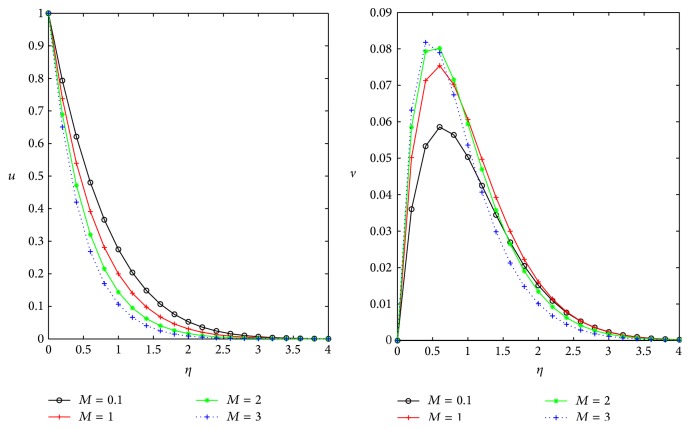
Velocities *u*, *v* with *M* for the cases *ϕ* = *D* = 0, *m* = 0.5, and *Ω* = 0.4.

First, we validate our analytical results with the numerical results of Rao et al. [22] by expressing primary and secondary velocities with magnetic field assuming the absence of inclination of the magnetic field and permeability of the porous medium in the system shown in Figure 2, for the case *ϕ* = *D* = 0 and fixing *t* = 1, *m* = 0.5, and *Ω* = 0.4. Our results in Figure 2 are identical results shown in Figure 3 of Rao et al. [22].

In the absence of *D*,  *ϕ* the governing equations of our problem is identical to Rao et al. [22] and they solved the governing equations using FEM technique, but we are presenting analytical solution for the extension of this problem.

**Figure 3:**
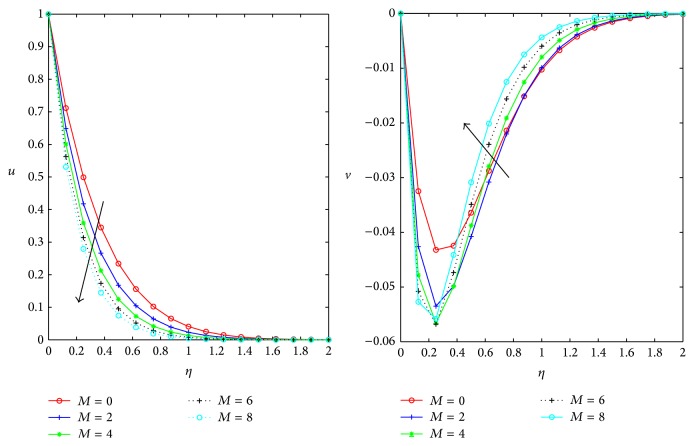
Influence of Hartman number *M* on *u*, *v* for *D* = *m* = 0.5, *ϕ* = *π*/4, and *Ω* = 0.4.

We have computed results for primary, secondary velocities and skin friction for fixed *M* = 1, *m* = 0.5, *Ω* = 0.4, *D* = 0.5, *t* = 1, and *ϕ* = *π*/4 different control parameters.  Figure 3 shows the variation of the primary *u* and secondary *v* velocities versus normal coordinate *η* with the Hartman number *M*. Primary velocity *u* decreases with increase in the Hartman number (*M*) in the boundary layer which shows the general tendency of a Lorentz force has oppose the motion of an electrically conducting fluid. But the magnitude of the secondary velocity *v* increases with increase in Hartman number within the thin boundary layer near the plate boundary and then flow that gets reversed behaves as a primary velocity in presence of magnetic field.

**Figure 4:**
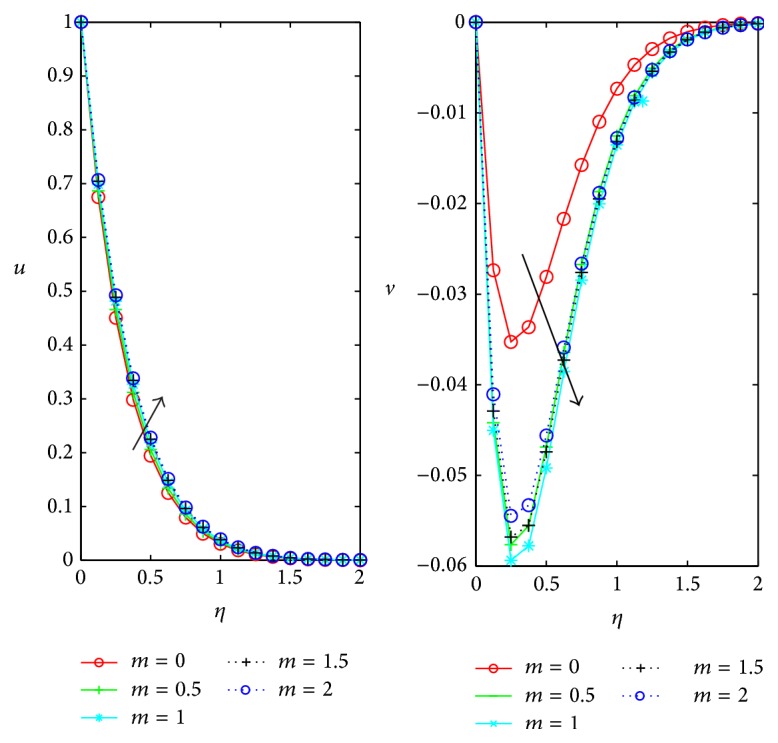
Influence of hall effect *m* on *u*, *v* for *D* = 0.5, *ϕ* = *π*/4, *M* = 1, and *Ω* = 0.4.

Figure 4 represents the variation of the primary *u* and secondary *v* velocities with *η* under the influence of the Hall current parameter *m*. We find that primary velocity *u* slightly increases with increase in *m* and as it moves away from the boundary, velocity tends to zero. Magnitude of the secondary velocity gets enhanced with the increase in Hall current parameter *m* for 0 ≤ *m* ≤ 1, but for further increase in *m* in the domain 1 < 1.5 ≤ *m* ≤ 2 secondary velocity *v* depreciates within the thin boundary layer near the plate.

**Figure 5:**
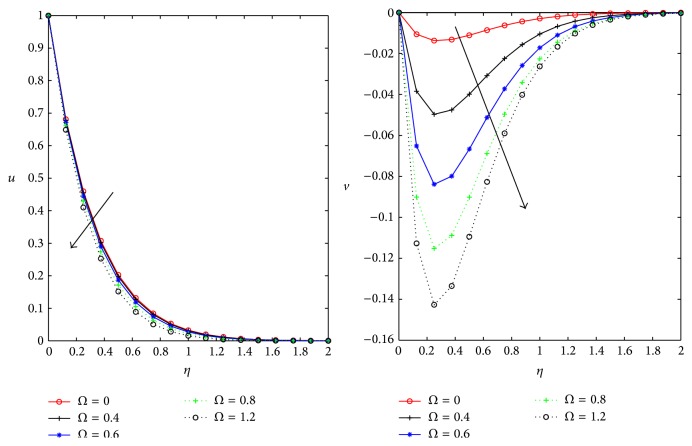
Influence of rotation *Ω* on *u*, *v* for *D* = *m* = 0.5, *ϕ* = *π*/4, and *M* = 1.

Figure 5 represents the variation of the primary and secondary flow velocities with *Ω* rotation of the fluid/plate boundary. In the absence of boundary rotation primary velocity is maximum and primary flow *u* depreciates with increase in the *Ω*, whereas secondary flow velocity *v* is monotonically increasing with the plate boundary rotation.

**Figure 6:**
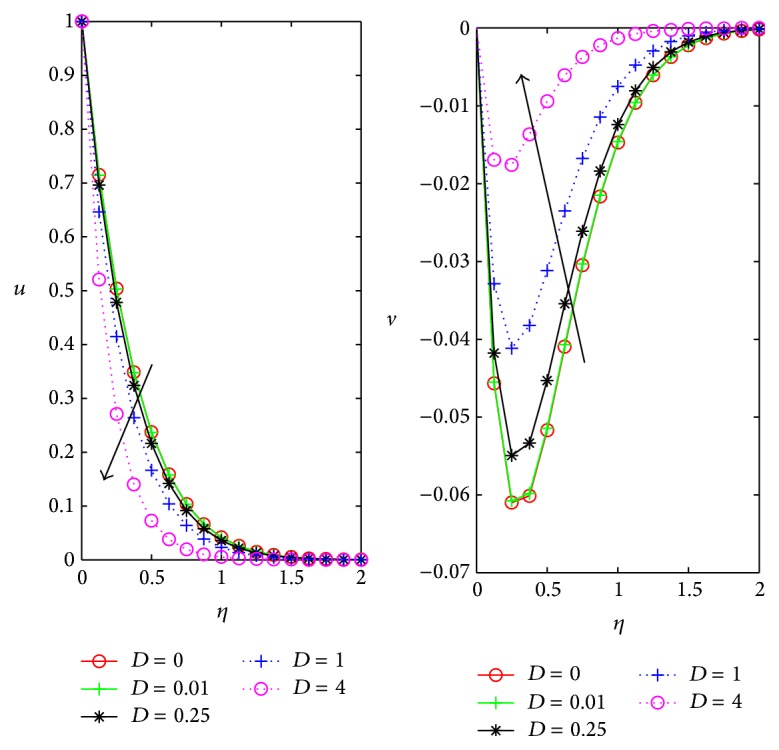
Influence of Darcy parameter *D* on *u*, *v* for *m* = 0.5, *ϕ* = *π*/4, *M* = 1, and *Ω* = 0.4.

Figure 6 represents the variation of the primary *u* and secondary flow *v* velocities with Darcy parameter *D*. In the absence of the Darcy parameter both primary and secondary velocities are maximum in magnitude and both velocities decrease with increase in Darcy parameter. Physically, it means that decrease in the permeability of the porous medium decreases both velocities.

**Figure 7:**
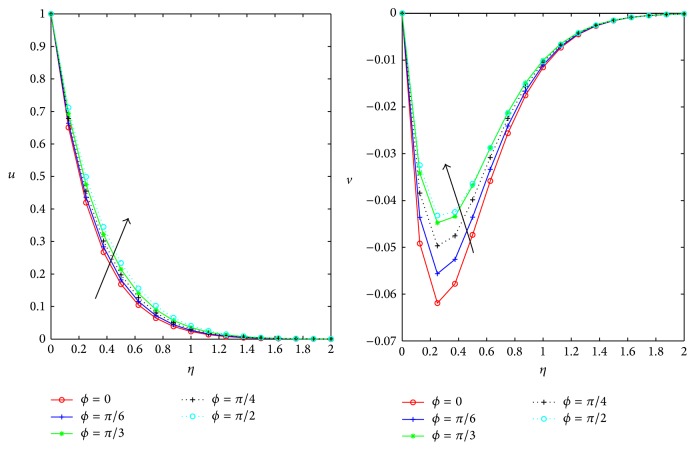
Influence of Inclination of magnetic field *ϕ* on *u*, *v* for *D* = *m* = 0.5, *M* = 1, and *Ω* = 0.4.

Figure 7 represents the variation of the primary secondary flow velocities with direction of the magnetic field parameter *ϕ*. Primary velocity increases as the direction of the magnetic field changes in the region 0 ≤ *ϕ* ≤ *π*/4; *π*/3 < *ϕ* ≤ *π*/2 but velocity slightly decreases in the region *π*/4 < *ϕ* ≤ *π*/3 of inclination of the magnetic field within the boundary layer region, whereas the magnitude of the secondary flow velocity *v* decreases gradually in the region 0 ≤ *ϕ* ≤ *π*/4; *π*/3 < *ϕ* ≤ *π*/2 of the direction of the inclined magnetic field, but slightly *v* increases in the region of inclination *π*/4 < *ϕ* ≤ *π*/3. So, in the absence of the inclination of the magnetic field the primary velocity is minimum and secondary velocity is maximum.

**Figure 8:**
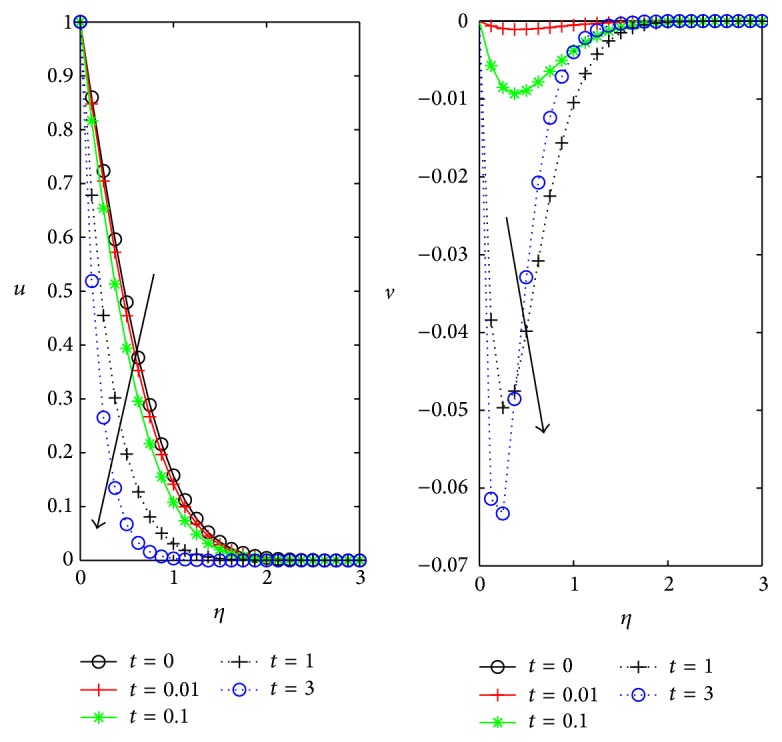
Influence of unsteadiness *t* on *u*, *v* for *D* = *m* = 0.5, *ϕ* = *π*/4, *M* = 1, and *Ω* = 0.4.

Figure 8 shows that in a small interval of time how primary and secondary flow velocities changes. At steady state condition primary velocity is maximum, as time *t* increases the velocity *u* decreases within the boundary layer region. Secondary flow velocity *v* increases gradually with time *t* ≤ 1, but for higher value *t* = 3 the secondary flow depreciates at dimensional distance *η* ≈ 0.5 from the plate. Secondary flow tends to exhibit the steady flow with increase in time with thin boundary layer.

Nondimensional viscous shear stresses *τ*_*x*_, *τ*_*y*_ (skin friction) at the boundary *z* = 0 due to the primary and secondary flows with control parameters are shown in Table 1. Components of the skin frictions *τ*_*x*_, *τ*_*y*_ acting on the surface increase with increase in magnetic field *M*. Hall currents show typical behavior on skin friction and *τ*_*x*_ decreases with increase in hall current parameter but *τ*_*y*_ increases with increase for 0 ≤ *m* ≤ 1 and then decreases for higher values of 1 < *m* ≤ 3. As there is increase in the rotation parameter *Ω* the skin frictional force *τ*_*x*_ decreases and *τ*_*y*_ increases. Viscous shear stresses *τ*_*x*_ increase with increase in the Darcy parameter 0 ≤ *D* ≤ 4, whereas *τ*_*y*_ increases with increase in Darcy parameter 0 ≤ *D* ≤ 1 and then *τ*_*y*_ decreases for higher Darcy value *D* = 4. That is, decrease in permeability of the porous medium skin friction *τ*_*x*_ increases gradually, whereas *τ*_*y*_ first increases and then decreases with increase in permeability of the porous medium.

In the absence of inclination of the magnetic field (*ϕ* = 0), that is, if we apply magnetic field normal to the plane *z* = 0 the skin frictions *τ*_*x*_, *τ*_*y*_ are maximum, but as the inclination *ϕ* increases from 0 < *ϕ* ≤ *π*/2 both skin frictions *τ*_*x*_, *τ*_*y*_ decrease. For small change in the time interval shear stress *τ*_*x*_ decreases greatly at same time as *τ*_*y*_ increases slightly.

## 5. Conclusions

We discuss the interplay between the Hartmann number *M*, rotation parameter (reciprocal of Ekman number) *Ω*, hall current parameter *m*, Darcy parameter *D*, and the direction of magnetic field on the primary and secondary flows, viscous shear stress at the plate by using the analytical solution obtained in the present study.

(i) Generally the magnetic field has a tendency to retard the flow and reduces the flow velocity, primary flow shows normal behavior whereas secondary flow velocity gets enhanced with increase in magnetic field within the thin Hartman-Ekman-Darcy boundary layer.
(ii) Hall current parameter shows less significance on the primary flow. But secondary flow velocity shows abnormality with Hall current exhibiting the flow is monotonically increasing for *m* ≤ 1 and then decreases monotonically for *m* ≥ 1.5.
(iii) Rotation of the fluid (rotating with same angular velocity as the system) reduces the primary velocity whereas it enhances the small scale secondary flow.
(iv) Primary and secondary flow decrease with increase in Darcy parameter indicating that decrease in permeability of the porous medium decreases flow.
(v) For change in the direction of the magnetic field 0 ≤ *ϕ* ≤ *π*/4, *π*/3 < *ϕ* ≤ *π*/2, primary flow increases and secondary flow velocity decreases except in the region *π*/4 < *ϕ* ≤ *π*/3. Changing the direction of the magnetic field can control flow field.
(vi) Lorentz force tends to resist the fluid flow, thus reducing skin friction.
(vii) Decrease in permeability of the porous medium skin friction *τ*_*x*_ increases gradually, whereas *τ*_*y*_ first increases and then decreases with increase in permeability.
(viii) Viscous shear stresses acting on the boundary is maximum when the direction of the magnetic field is perpendicular to the planar flow. Skin friction decreases with change in the direction (0 ≤ *ϕ* ≤ *π*/2) of the magnetic field.

## Figures and Tables

**Table 1:** Skin frictions (τ_*x*_, τ_*y*_) at the boundary *z* = 0.

*M*	*m*	Ω	*D*	ϕ	*t*	τ_*x*_	τ_*y*_
*0 *	0.5	0.4	0.5	π/4	1	2.0795	0.0893
*0.1 *	0.5	0.4	0.5	π/4	1	2.0893	0.095
*1 *	0.5	0.4	0.5	π/4	1	2.1874	0.1468
*2 *	0.5	0.4	0.5	π/4	1	2.3106	0.2002
*4 *	0.5	0.4	0.5	π/4	1	2.5704	0.2868
*8 *	0.5	0.4	0.5	π/4	1	3.0528	0.395
1	*0 *	0.4	0.5	π/4	1	2.2064	0.1055
1	*1 *	0.4	0.5	π/4	1	2.1542	0.1645
1	*1.5 *	0.4	0.5	π/4	1	2.1268	0.1640
1	*2 *	0.4	0.5	π/4	1	2.1090	0.1568
1	*3 *	0.4	0.5	π/4	1	2.0916	0.1413
1	0.5	*0 *	0.5	π/4	1	2.1954	0.0406
1	0.5	*0.1 *	0.5	π/4	1	2.1942	0.0666
1	0.5	*0.8 *	0.5	π/4	1	2.1733	0.2621
1	0.5	*1 *	0.5	π/4	1	2.1656	0.3246
1	0.5	*2 *	0.5	π/4	1	2.1567	0.6931
1	0.5	0.4	*0 *	π/4	1	2.0573	0.1232
1	0.5	0.4	*0.01 *	π/4	1	2.0597	0.1239
1	0.5	0.4	*0.25 *	π/4	1	2.1203	0.1374
1	0.5	0.4	*1 *	π/4	1	2.328	0.1555
1	0.5	0.4	*4 *	π/4	1	3.1171	0.1288
1	0.5	0.4	0.5	*0 *	1	2.2836	0.2242
1	0.5	0.4	0.5	*π*/6	1	2.237	0.1843
1	0.5	0.4	0.5	*π*/3	1	2.135	0.1136
1	0.5	0.4	0.5	*π*/2	1	2.0795	0.0893
1	0.5	0.4	0.5	π/4	*0.01 *	11.012	0.0072
1	0.5	0.4	0.5	π/4	*0.1 *	4.2029	0.0265
1	0.5	0.4	0.5	π/4	*1 *	2.1874	0.1468
1	0.5	0.4	0.5	π/4	*2 *	2.0315	0.2248
